# Supplementary material for: Post-mortem molecular profiling of three psychiatric disorders
Source: Genome Med. 2017 Jul 28;9:72. doi: 10.1186/s13073-017-0458-5 (PMC5534072; doi:10.1186/s13073-017-0458-5)
Supplement: Supplementary file 1 — Supplemental text with detailed methods. (DOCX 134 kb) [file 13073_2017_458_MOESM1_ESM.docx]

**Bowling et al.: Supplementary Information**

Supplemental Tables can be found as a separate file.

Supplemental Methods

**Sample Inclusion Criteria**

Psychological autopsies are completed on all samples prior to inclusion in the study. The primary purpose of the psychological autopsies is to classify decedents.  Classifications involve an extensive analysis of available data from multiple sources, including the medical examiner's conclusions, coroner's investigation, medical records, psychiatric records, toxicology results and 141-item family interviews.  Data from all or any combination of these sources are examined for information concerning details of death, psychopathology, symptom profile, substance use, physical health, medication use, educational background, employment history, and family history of psychiatric disorders and suicide.  For inclusion in this study, diagnosed samples were confirmed to suffer from MDD, BPD or SZ.  Controls were free of psychiatric illnesses and had no history of mental illness among first and second-order relatives.  All samples received independent diagnostic reviews to ensure agreement between clinical psychologist and psychiatrist.

**Metabolomics**

*Sample preparation*

Sections of approximately 100mg of frozen tissue were weighed and homogenized for 45 seconds at 6.5M/s with ceramic beads in 1mL of 50% methanol using the MP FastPrep-24 homogenizer (MP Biomedicals). We immediately returned the samples to ice on a shaker table at 8^o^C for 30 minutes. Samples were then incubated at -20^o^C for one hour and centrifuged at 12000 rpm for 10 min. Supernatant was stored at -80^o^C until analysis. Tissue weights measured before homogenization were used to normalize input amount. A sample volume equivalent to 10mg of initial tissue weight (i.e. 100 μL extract for 100mg starting tissue) was dried down at 55^o^C for 60 minutes using a vacuum concentrator system (Labconco). We derivatized the samples by methyoximation, adding 30μL of a 20mg/mL solution of methoxyamine in pyridine to the dried sample and incubating at 30^o^C for 30 minutes. Next, we completed trimethylsilylation adding 70μL MSTFA with 1% TCMS (Restek) and incubated at 60^o^C for 60 minutes.

We analyzed technical replicates of each tissue sample, in randomized order.

*GCxGC-TOFMS analysis*

All derivatized samples were analyzed on a Leco Pegasus 4D system (GCxGC-TOFMS), controlled by the ChromaTof software. The primary column is a 20m x 0.25mm x 0.5μm with RTX-5MS film (Restek), and the secondary column is 2.5m x 0.18mm x 0.2μm RTX-200MS film (Restek). We analyzed 1μL injections in split mode using a split ratio of 1:10. Both the inlet and transfer line temperatures were 280**°**C. The helium (carrier gas) flow rate was 1mL/min. Initial oven temperatures were 60**°**C for the primary oven and 80**°**C for the secondary oven, with the modulator temperature maintained at 15**°**C above the primary oven temperature. After a 1 minute hold, all oven temperatures were increased by 6^o^C/min for 18 minutes, then 5^o^C/min for 11 minutes, and finally 10^o^C/min for 8 minutes when the primary oven reached 300^o^C where it held for 12 minutes. The modulation time for the second dimension was 5s with a 0.4s hot pulse and a 2.1s cold pulse. The ion source temperature was 250**°**C. Data were collected at 100 spectra/second after a 7-minute solvent delay. The total run time for each sample was approximately 45 minutes.

*Data analysis and metabolite identification*

We used ChromaTOF 4.5 software (Leco) for peak calling and deconvolution. Peaks were identified by spectral match using the NIST, GOLM, and Fiehn libraries (Leco), and confirmed by running derivatized standards (Sigma).

To facilitate our untargeted metabolomic analysis, we created a reference that ChromaTOF used to compare each sample: sample peaks that match a reference peak are assigned the corresponding QuantMass to match the reference peak. We then used the software package Guineu v1.0 to align common variables among the individual sample files. Guineu parameters were: RT Lax 20.0, RT2 Lax 1.0, RT Penalty 25.0, RT2 Penalty 20.0, RTI penalty 0.0, Minimum Spectrum Match 0.5, Bonus for matching names 20.0, Drop peaks with similarity less than 500.0. The output is a multi-way alignment merging all sample files to a single file with a relative metabolite quantitation for each sample. An in-house script takes the output from Guineu, and further combines any peaks meeting the requirements below that have not been combined by Guineu. This script takes into account peak name, change in primary and secondary retention time (RT1 and RT2), and a rudimentary spectral "match"--counting the fraction of the ten most ions that match. Peaks are combined under either of the following circumstances: 1) deltaRT1<2, deltaRT2<0.1, and match of at least 7 of 10; 2) identical names, RT1<5 and RT2<0.1 or 3) RT1<5, RT2<0.1 and match of at least 8 of 10. On average this in-house script reduces the total number of peaks by approximately 25%. Peaks present in less than two-thirds of samples were excluded from further analysis. Sample replicates were averaged. Peak areas were sum normalized prior to comparisons, missing values were replaced using the K-nearest neighbor method, and the data was auto-scaled (mean-centered, divided by the standard deviation for each metabolite) using the web application MetaboAnalyst 3.0 (2). Additional statistical analyses were performed in R.

1. Aurbach E, Inui E, Turner C, Hagenauer M, Prater K, Li J, Absher D, Shah N, Akil H: Fibroblast Growth Factor 9 is a Novel Modulator of Negative Affect. PNAS 2015; In press

2. Xia J, Sinelnikov I V., Han B, Wishart DS: MetaboAnalyst 3.0--making metabolomics more meaningful [Internet]. Nucleic Acids Res. 2015; gkv380–[cited 2015 Apr 21] Available from: http://nar.oxfordjournals.org/content/early/2015/04/20/nar.gkv380.full
