# Supplementary material for: Post-mortem molecular profiling of three psychiatric disorders
Source: Genome Med. 2017 Jul 28;9:72. doi: 10.1186/s13073-017-0458-5 (PMC5534072; doi:10.1186/s13073-017-0458-5)
Supplement: Supplementary file 3 — Supplemental figures S1–S7. (PDF 524 kb) [file 13073_2017_458_MOESM3_ESM.pdf]

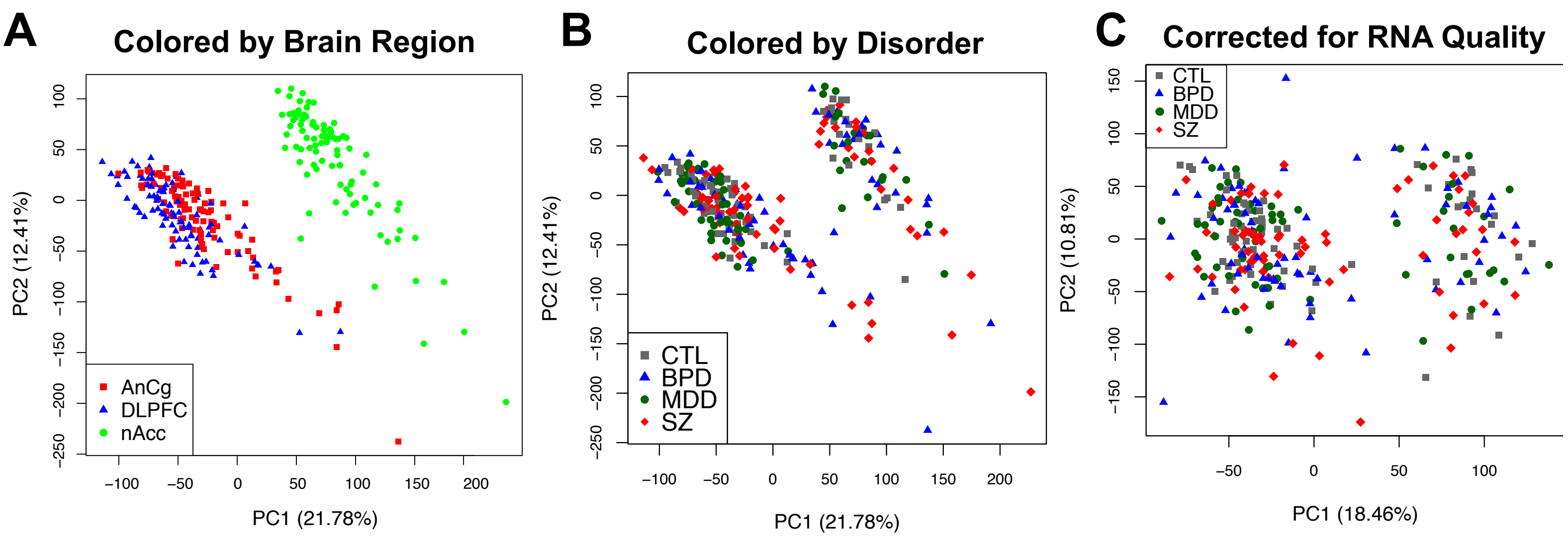

Figure S1. A) Principal components analysis of all 281 brain tissues. AnCg (red squares), DLPFC (blue triangles), nAcc (green circles). B) Principal components analysis of all 281 brain tissues. CTL (gray squares), BPD (blue triangles), MDD (green circles), SZ (red triangles). (C) Principal components analysis of all 281 brain tissues after correcting RNA-seq data for alignment quality. CTL (gray squares), BPD (blue triangles), MDD (green circles), SZ (red triangles).

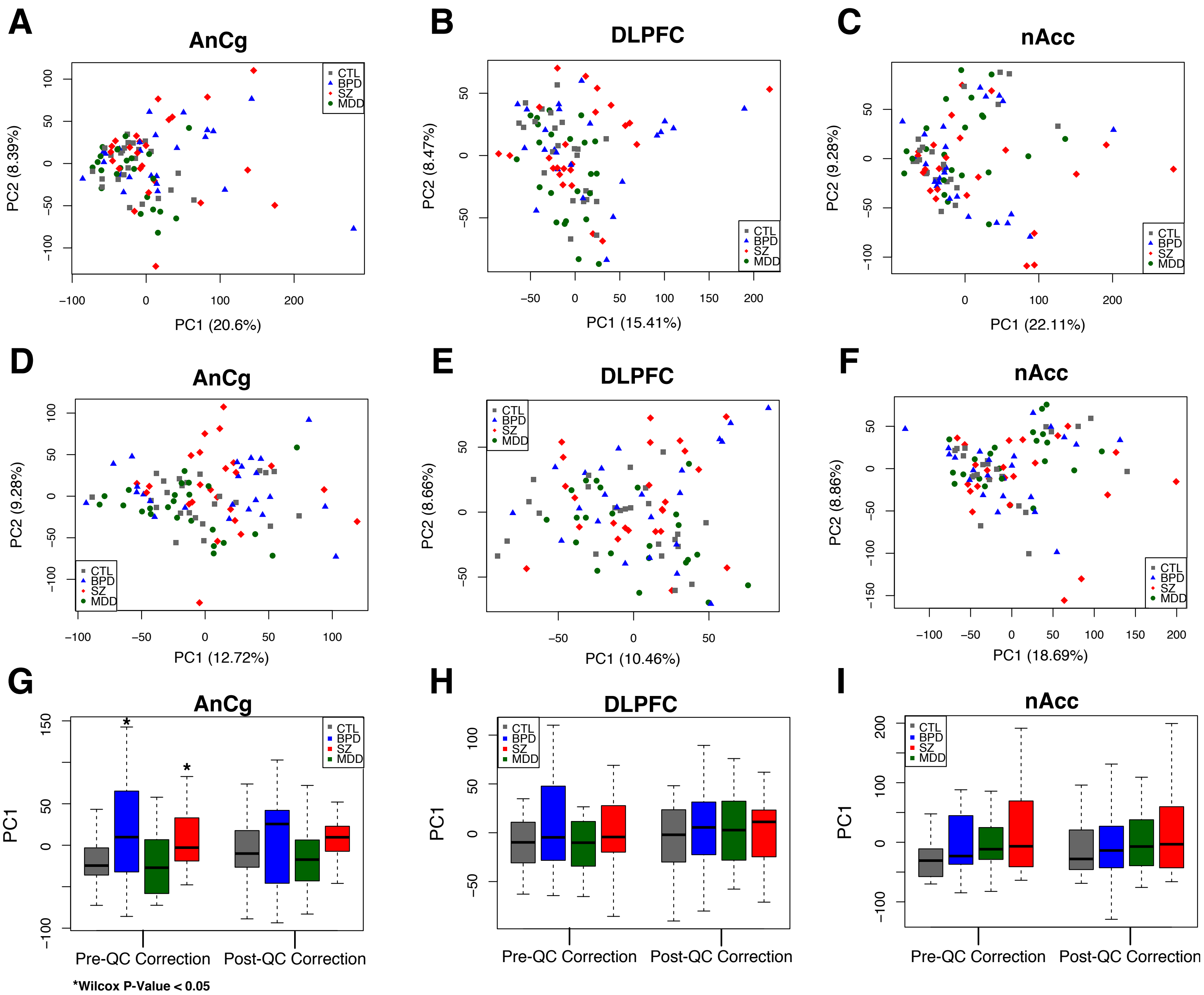

Figure S2. Principal components analysis of all AnCg (A,D), DLPFC (B,E), and nAcc (C,F) samples before (A-C) and after (D-F) correction for RNA-seq alignment quality. (G-I) PC1 values in CTL (gray), BPD (blue), MDD (green), and SZ (red) patients pre- and post-RNA-seq alignment quality correction in the AnCg (G), DLPFC (H), and nAcc (I).

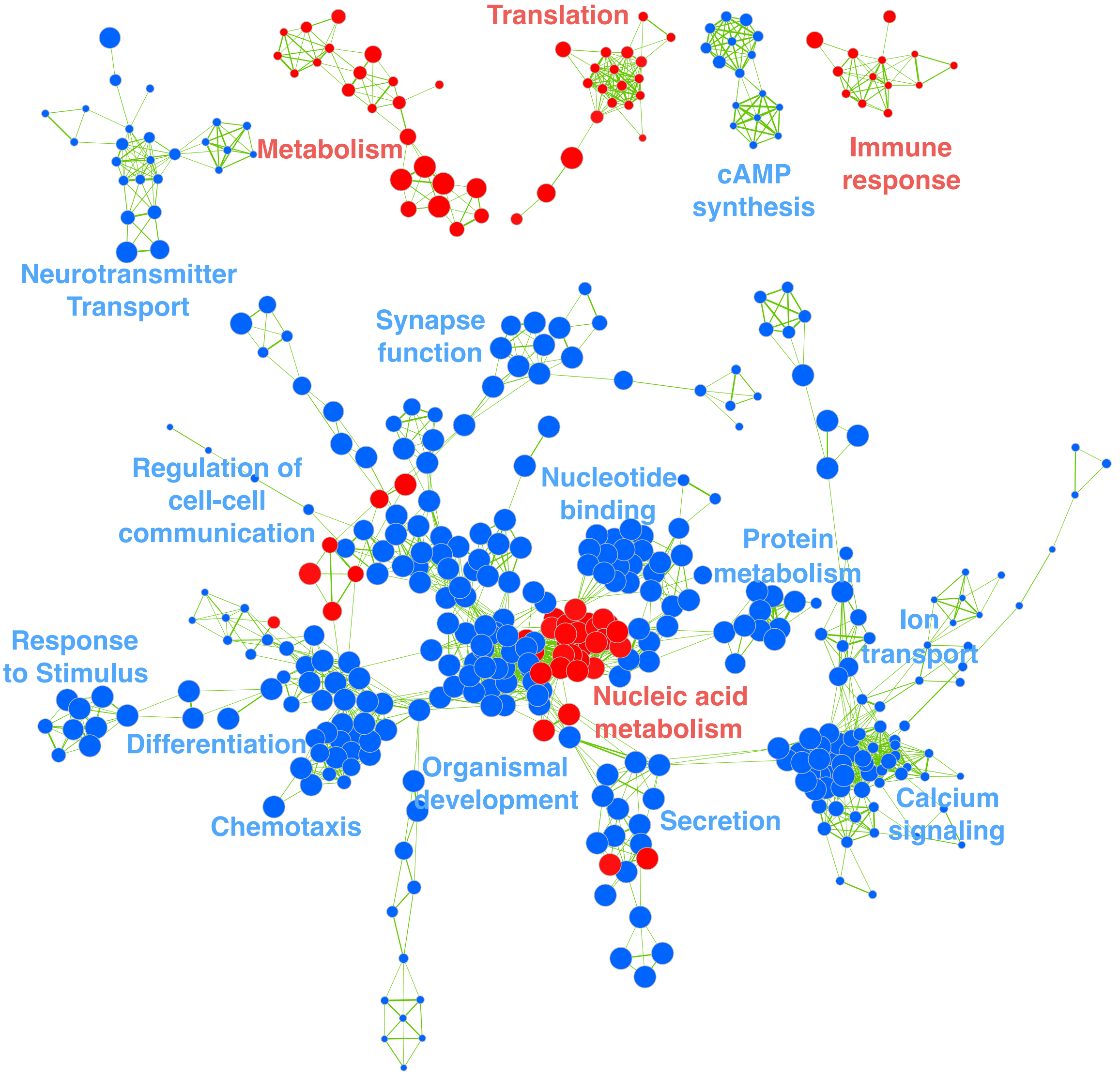

Figure S3. GO-term analysis for genes differentially expressed in SZ vs. CTL in AnCg (FDR<0.05). Up-regulation (red circles), down-regulation (blue circles).

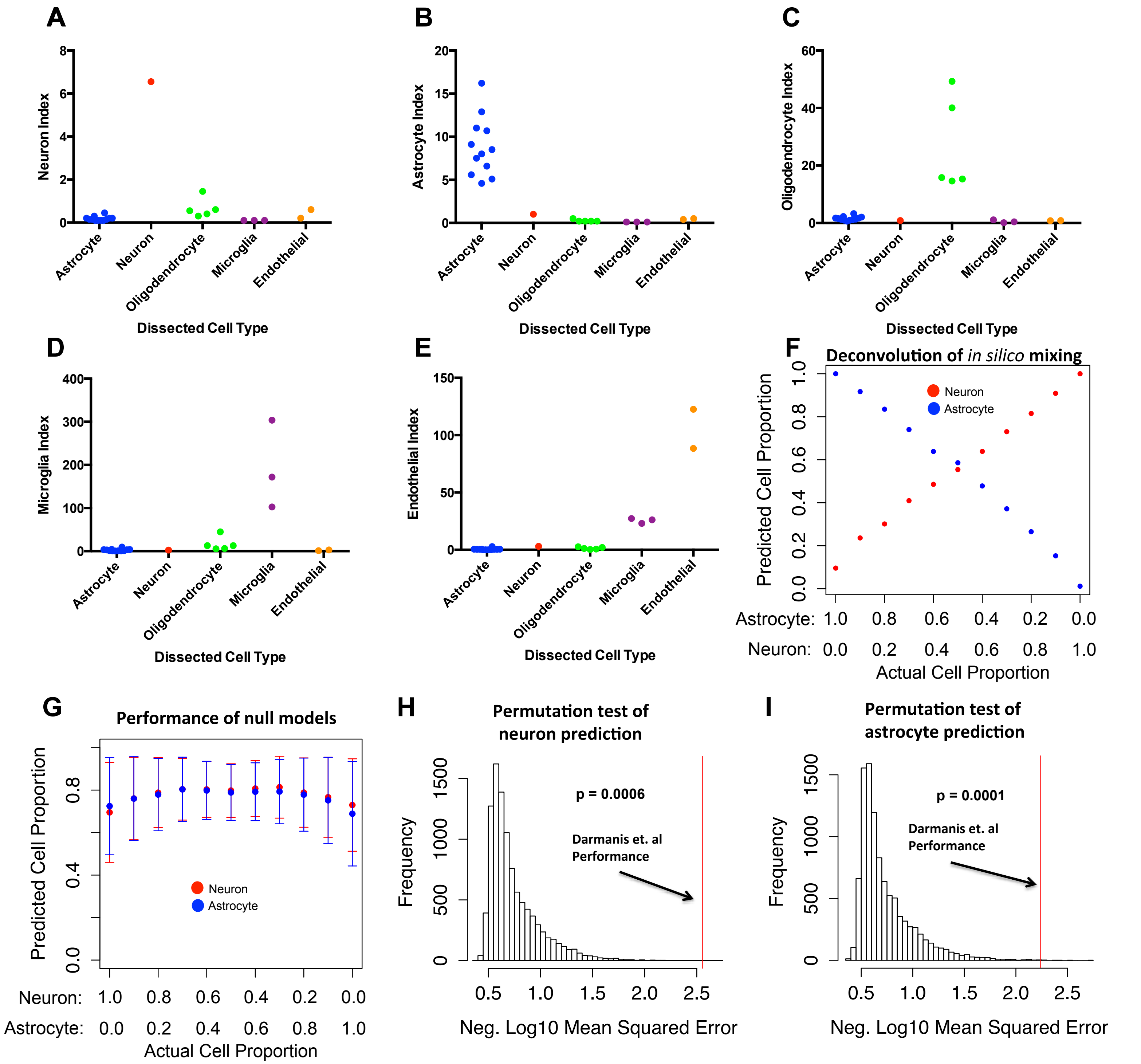

Figure S4. Using cell-type specific index based on single cell sequencing, we calculated the index for purified populations of (A) neurons, (B) astrocytes, (C) oligodendrocytes, (D) microglia, and (E) endothelial cells. In all cases the calculated indexes were specific to the purified population (F) Neuron and astrocyte indices were used to predict the proportion of each cell type in in silico mixed cell-type populations. (G) Compared to the Darmanis et al. indexes, 10,000 randomly generated gene sets do not predict cell type proportions. Mean values with standard deviation are plotted (H, I) Histogram of mean squared error of null index cell type proportion predictions for mixed neuron and astrocyte transcriptomes with Darmanis et al. gene performance indicated in red. The Darmanis gene set far outperforms any randomly generated gene sets.

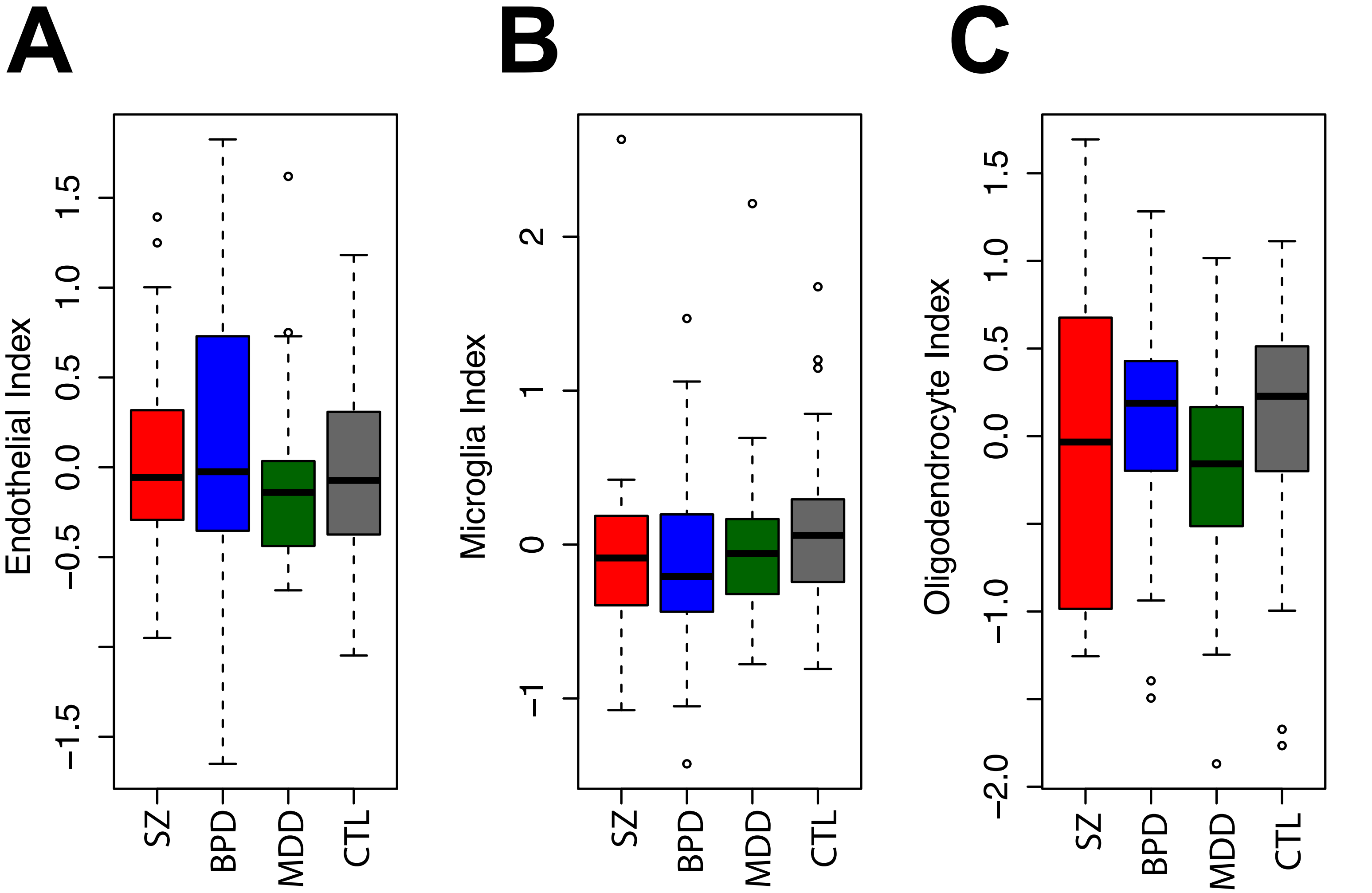

Figure S5. Boxplots of endothelial (A), microglia (B), and oligodendrocyte (C) cell type indices in SZ (red), BPD (blue), MDD (green), and CTL (gray) individuals using indices derived from Darmanis et al.

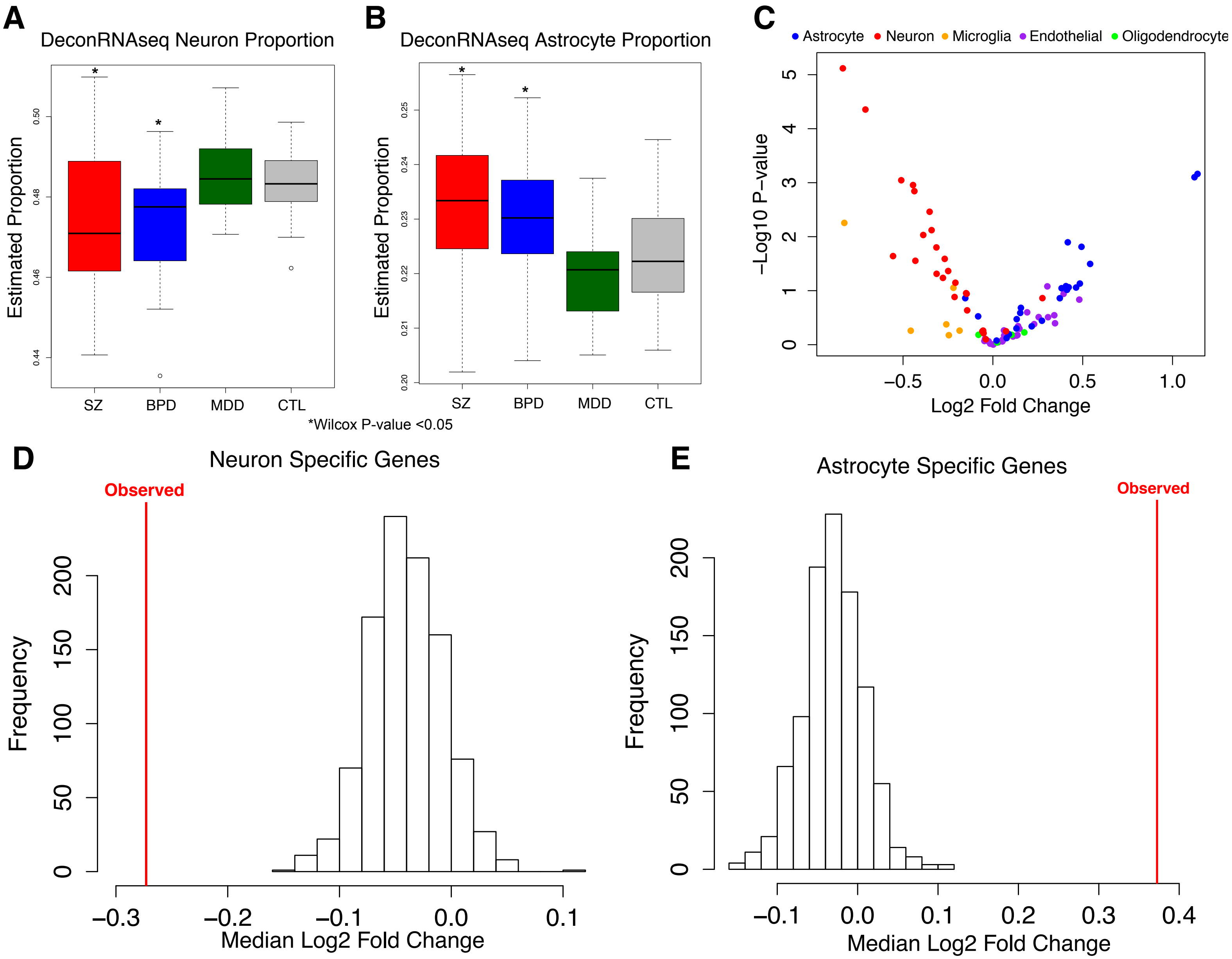

Figure S6. (A,B) Estimated neuron (A) and astrocyte (B) cell type proportions using the deconRNAseq deconvolution algorithm in SZ, BPD, MDD, and CTL individuals. (C) Volcano plot of cell type specific transcripts in the Darmanis et al. gene sets show altered expression in SZ v CTL. Neuronal transcripts are enriched for loss of expression and astrocyte-specific transcripts are enriched for increased expression (D,E) Histograms representing the distribution of median log2 fold change for expression level matched gene sets to neuron (D) and astrocyte (C) specific genes. Red lines indicate median log2 fold change observed for neuron- and astrocyte-specific genes respectively.

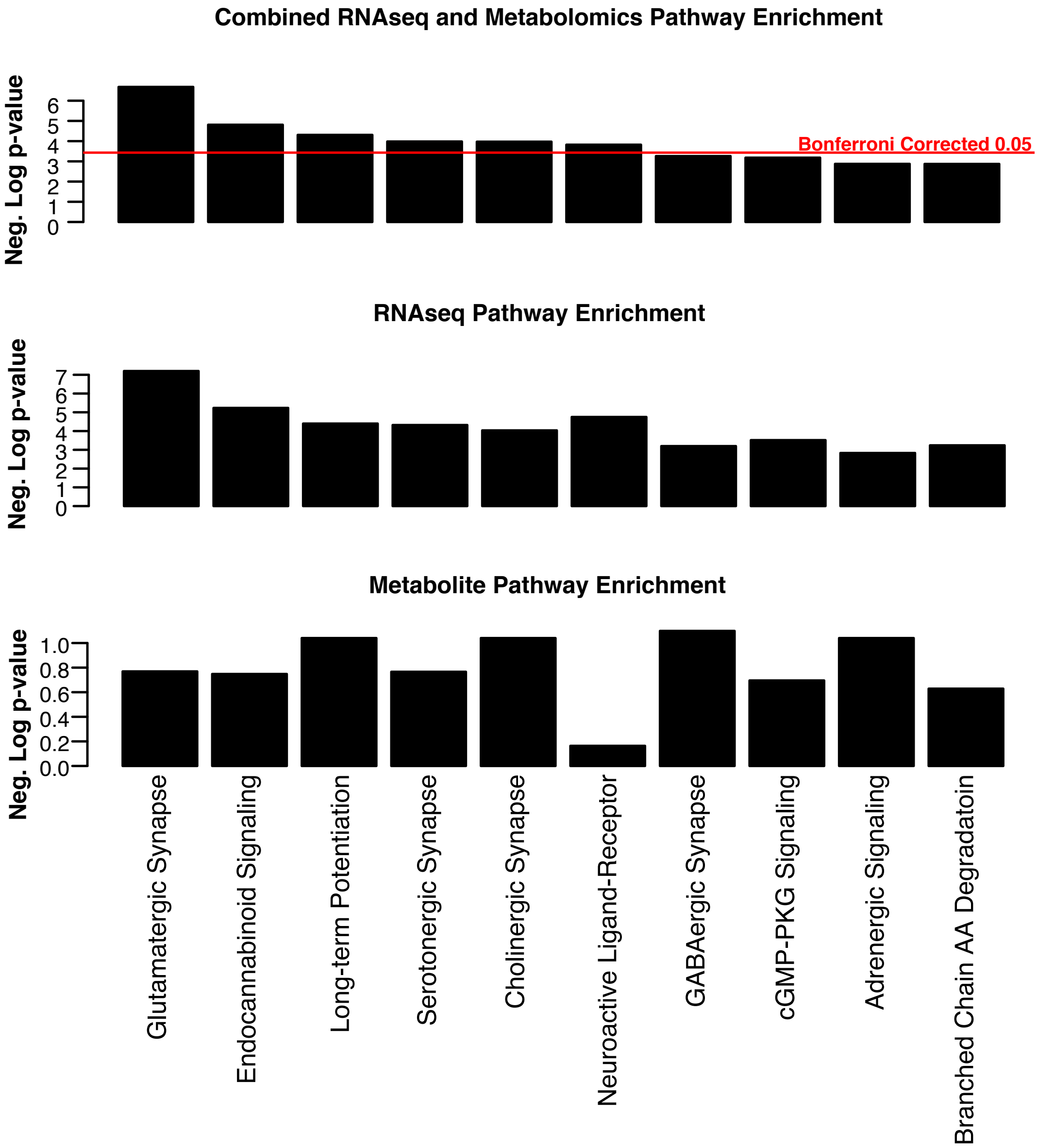

Figure S7. Integrated KEGG pathway analysis of metabolite and RNA-seq differences between SZ and CTL patients. Top 10 pathways are shown for metabolite, gene and combined analysis.
